# The in vitro cytotoxic, genotoxic, and oxidative damage potentials of the oral artificial sweetener aspartame on cultured human blood cells

**DOI:** 10.3906/sag-2001-113

**Published:** 2020-04-09

**Authors:** Kenan ÇADIRCI, Özlem ÖZDEMİR TOZLU, Hasan TÜRKEZ, Adil MARDİNOĞLU

**Affiliations:** 1 Department of Internal Medicine, Erzurum Regional Training and Research Hospital, Health Sciences University, Erzurum Turkey; 2 Department of Molecular Biology and Genetics, Faculty of Science, Erzurum Technical University, Erzurum Turkey; 3 Department of Medical Biology, Faculty of Medicine, Atatürk University, Erzurum Turkey; 4 Department of Pharmacy, University “G. d’Annunzio” Chieti-Pescara, Chieti Italy; 5 Science for Life Laboratory, KTH-Royal Institute of Technology, Stockholm, SE-17121 Sweden; 6 Centre for Host-Microbiome Interactions, Faculty of Dentistry,Oral & Craniofacial Sciences, King’s College London, London, SE19RT United Kingdom

**Keywords:** Aspartame, cytotoxicity, genotoxicity, antioxidant activity, human whole blood cultures

## Abstract

**Background/aim:**

Aspartame (APM, L-aspartyl-L-phenylalanine methylester) is a low-calorie, nonsaccharide artificial sweetener widely used in foods and beverages. When metabolized by the body, APM is broken down into aspartic acid, phenylalanine amino acids, and a third substance, methanol. Since the amino acid phenylalanine serves as a neurotransmitter building block affecting the brain, and methanol is converted into toxic formaldehyde, APM has deleterious effects on the body and brain. Thus, its safety and, toxicity have been the subjects of concern ever since it was first discovered. Although many studies have been performed on it, due to the presence of conflicting data in the literature, there are still numerous question marks concerning APM. Therefore, the safety of aspartame was tested using in vitro methods.

**Materials and methods:**

We aimed to evaluate the in vitro cytotoxic effects by using 3-(4,5-dimetylthiazol-2-yl)-2,5-diphenyltetrazolium bromide (MTT) and lactate dehydrogenase release tests, genotoxic damage potential by using chromosome aberration (CA) assay, and antioxidant/oxidant activity by using total antioxidant capacity (TAC) and total oxidative stress (TOS) analysis in primary human whole blood cell cultures.

**Results:**

The results of the MTT test showed that APM led to significant decreases in cell viability in a clear concentration-dependent manner. Moreover, an increase in CA frequency was found in the cells treated with APM. However, APM treatments did not cause any significant changes in TAC and TOS levels in whole blood cultures.

**Conclusion:**

Overall, the obtained results showed that APM had genotoxicity potential and a concentration-dependent cytotoxic activity in human blood cells.

## 1. Introduction

High intensity-sweeteners (HIS) are natural, semi-synthetic, or synthetic chemical substances used as an alternative to sugar in food products, beverages, and some oral medications. Aspartame (APM), a member of the semi-synthetic sweetener group, was first discovered incidentally in 1965 by the chemist James M. Schlatter during research into anti-ulcer drugs [1,2]. APM was approved as an artificial sweetener by the Food and Drug Administration (FDA) in 1981 [3], and is used in more than 6000 products, in non-alcoholic beverages, chewing gum, sugar, yoghurt, and some pharmacological products such as sugar-free cough drops [4]. Following its approval by the FDA, APM has become a sweetener increasingly used in both diabetics and obese patients on weight loss programs, and also in healthy individuals as a food additive.

At the reports of American Dietetic Association, World Health Organization (WHO) and Food and Agriculture of the United Nations have determined an acceptable daily intake (ADI) as a conservative estimate that can be used as a safety marker for artificial sweeteners**.** In 2005, the FDA set that value as 50 mg/kg for APM, while in 2006 European Food Safety Authority (EFSA, 2006) set it as 40 mg/kg [5]. Following oral intake, APM is absorbed by the intestinal lumen and hydrolyzed into phenylalanine (50%), aspartic acid, an excitatory amino acid (40%), and methanol (10%) [6]. It acquires its sweetness from T1R2 and T1R3, from the T1R class receptor family, a G protein-coupled receptor family [7].

Chronic exposure to APM, one of the six generally recognized as safe (GRAS) non-nutritive sweeteners (NNS) recognized by the FDA in the USA (saccharine, aspartame, sucralose, neotame, acesulfame-K, and stevia), has been reported to cause neurological disorders such as headache, eye problems including blurred vision, insomnia, torpor, memory loss, nausea, speech impairment, personality changes, loss of energy, hyperactivity, and hearing problems [8,9]. The data in the literature concerning the molecule APM, regarded as safe and approved for use by the FDA, are inconsistent, though some show side-effects associated with APM use. Doubts concerning the use of the APM molecule persist to this day due to reports that it can cause neurological damage and induce apoptosis by adversely affecting the oxygen balance in brain [10], degenerative damage in nerve cells [11], liver and kidney damages via increased oxidative stress [6,9,12] and also cancer development due to its carcinogenic effects on the entire body [4,13]. To the best of our knowledge no study has been conducted to investigate the cytotoxic, genotoxic, and oxidative potentials of APM on human blood cells. Therefore, in the present study, in vitro biological activities of APM were determined via measuring cytotoxicity, genotoxicity, and oxidative damage levels in cultured human blood cells for the first time.

## 2. Methods

### 2.1. MTT assay

Whole heparinized human blood samples (10 mL) was collected from five healthy, male, non-smoking donors between the ages of 22 and 25 years, with no history of exposure to any genotoxic agent. Peripheral blood mononuclear cells (PBMC) were collected by centrifugation on Ficoll-Hypaque gradient (Cambrex; 800 g, 20 min, without brake) and placed in a 96-well plate containing the growth medium. The cells were treated with different concentrations (3.125, 6.25, 12.5, 25, 50, and 100 mg/L) of APM for 48 h. At the end of incubation, 5 mg/mL MTT solution was added to each well and incubated for 4 h. To solubilize formazan crystals, dimethyl sulfoxide was added to each well and the absorbance was measured at 570 nm using a spectrophotometer (Synergy-HT; BioTek Winooski, VT, USA). Triton X-100 treated cells were used as positive control and the cells without any treatment were used as a negative control.

### 2.2. Lactate dehydrogenase (LDH) release assay

PBMC were placed in a 96-well plate containing the growth medium and exposed to different concentrations (3.125, 6.25, 12.5, 25, 50, and 100 mg/L) of APM for 48 h. After the incubation period, LDH assay was carried out using a commercial kit (CytoSelectTM LDH Cytotoxicity Assay kit) according to the provider’s instructions. Briefly, 90 µL of the supernatant from each well was mixed with 10 μL of LDH reagent and incubated at 37 °C for 30 min. The optical density was determined at 450 nm using a spectrophotometer (Synergy-HT; BioTek Winooski, VT, USA). Triton X-100-treated cells were used as positive control and the cells without any treatment were used as a negative control.

### 2.3. Total antioxidant capacity (TAC) and total oxidant status (TOS) assays

PBMC was seeded in a 48-well plate and treated with various concentrations (3.125, 6.25, 12.5, 25, 50, and 100 mg/L) of APM for 48 h at 37 °C with 5% CO2. Then, plasma samples were collected to perform TAC (Rel Assay Diagnostics®,Turkey) and TOS assay Rel Assay Diagnostics®, according to the provider’s guide.

### 2.4. CA assay

Human blood cells were treated with APM at IC50 concentration and cultured for 72 h in an incubator. Colchicine (0.02 μg/mL) was added to the samples 2 h before the harvest of cells. At the end of 72 h, the cells were collected by centrifugation after hypotonic treatment (0.075 M KCl) and fixation with methanol/acetic acid. The fixed-cell suspension was dropped on glass slides. Then, air-dried slides were stained with Giemsa in phosphate buffer (pH 6.8). Fifty well-spread metaphases were counted and scored for chromosome aberration according to the Environmental Health Criteria 46 for environmental monitoring of human populations (IPCS, 1985).

### 2.5. Ethics

The study was approved by the local Ethics Committee (2019/03-29) and was in accordance with the Declaration of Helsinki and the International Conference on Harmonization for Good Clinical Practice. Written informed consent was obtained from all patients.

### 2.6. Statistical analysis

Statistical analysis was performed using SPSS software (version 18.0, SPSS, Chicago, IL, USA). Duncan’s test was used for the statistical analysis of experimental values in the MTT, LDH, CA, TAC, and TOS analysis. Statistical decisions were made with a significance level of 0.05.

## 3. Results

### 3.1. Cytotoxicity assays

**Figure 1:**
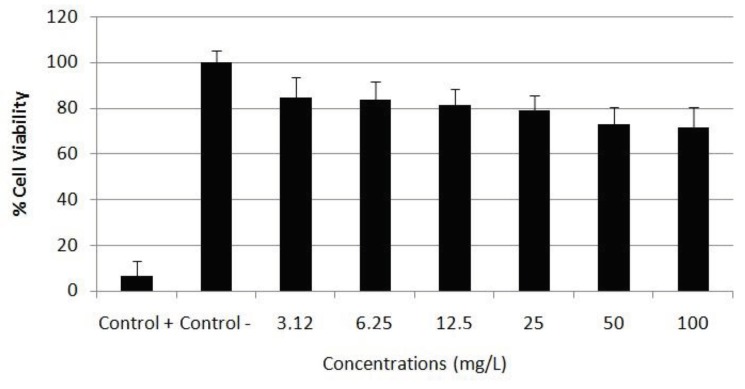
Viability of human blood cells after 48 h of exposure to 0–100 mg/L APM. Control (-): negative control; Control (+): positive control.

**Figure 2:**
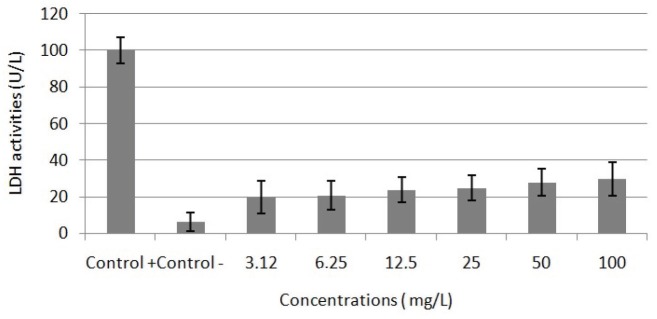
LDH activities in cultured human blood cells treated with different concentrations (0–100 mg/L) of APM for 48 h. The abbreviations are the same as those in Figure 1.

After treatments with different concentrations of APM for 48 h, cytotoxicity was examined using colorimetric and enzymatic methods including MTT and LDH assays. The results of MTT assay are presented in Figure 1. According to the obtained results, APM treatment showed a low impact on cell viability but in a concentration-dependent manner. The LDH activities of different concentrations of APM on blood cells are represented in Figure 2. The obtained results are similar to MTT assay results. The IC50 value of APM on lymphocyte cells was calculated according using MTT assay results as 287.342 mg/L.

### 3.2. Antioxidative/oxidative assays

**Figure 3:**
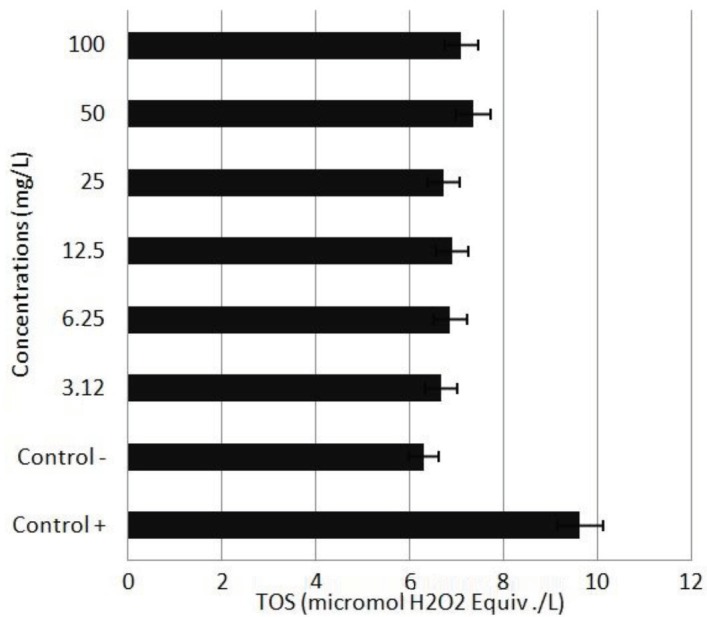
TOS levels in cultured human peripheral blood cells exposed to APM for 48 h. The abbreviations are the same as those in Figure 1.

**Figure 4:**
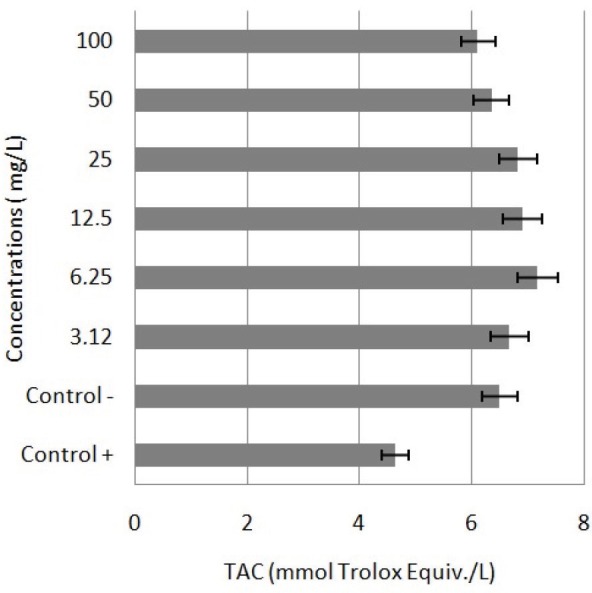
TAC levels in cultured human peripheral blood cells exposed to APM for 48 h. The abbreviations are the same as those in Figure 1.

To investigate the potential antioxidative/oxidative effects of APM on human blood cells, TAC and TOS assays were used to determine total antioxidant and oxidant levels. According to the TAC assay results, the highest TAC level was observed in the cells treated with 6.25 mg/L APM. On the other side, concentrations lower than 50 mg/L of APM did not cause significant alteration in TOS level. When compared to the control group, 50 and 100 mg/L of APM increased the TOS levels. The results of TOS levels are presented in Figure 3 and the results of TAC levels in Figure 4.

**Figure 5:**
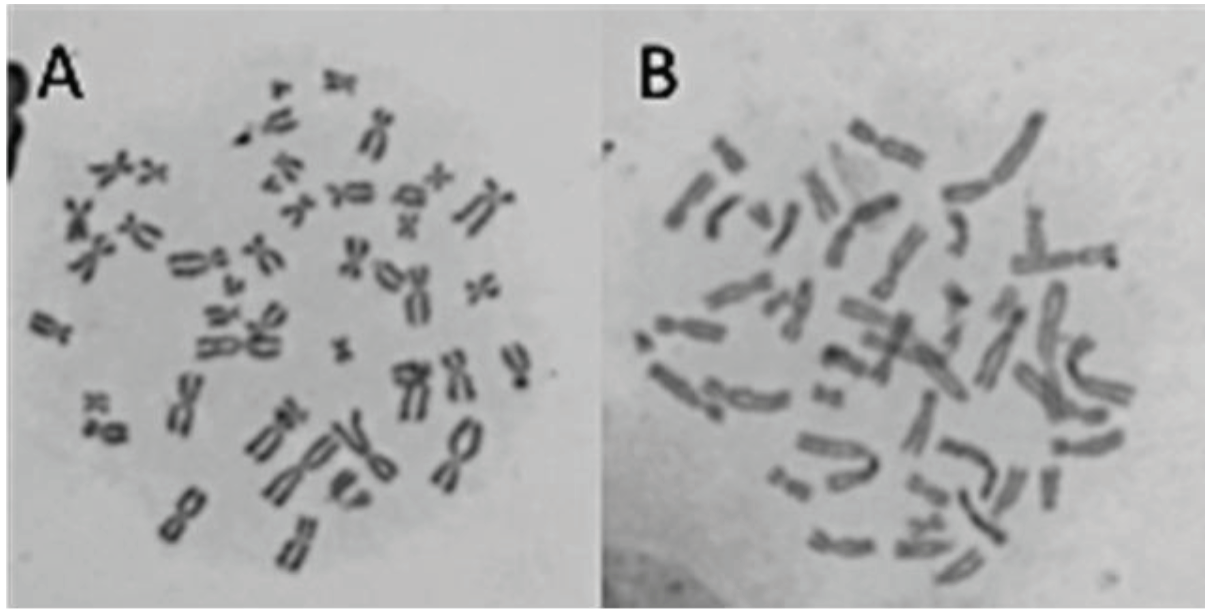
Representative images of chromosomal aberrations observed in cultured human peripheral blood cells. (A: Control group, B: Treatment with APM in IC50 dose).

### 3.3. Genotoxicity assays

u2941 5 shows the results of CA assay in lymphocytes. Based on the results, CA frequency is 0.24, 0.68, and 0.43 in negative control group, positive control group, and APM-treated group, respectively. Mitomycin C used as a positive control caused significant increases in CA frequencies. Moreover, APM at IC50 concentration induced a considerable increase in formations of CA when compared to control group.

## 4. Discussion

APM is rapidly and completely metabolized into its amino acid components (aspartic acid and phenylalanine) and methanol following absorption. Studies have shown that rapidly metabolized APM is dispersed throughout the body, particularly in organs with rapid protein manufacture and breakdown [14]. The APM molecule has always been the subject of research into its safety and toxicity due to the presence in neurotransmitters acting on the brain of phenylalanine resulting from APM metabolism being capable of leading to neurobiochemical and behavioral changes [15], and to the conversion of methanol to toxic formic acid.

Methanol and its short-lived oxidized product formaldehyde are compounds naturally present in normal healthy individuals. Sources of human physiological methanol include fruit and vegetables, the fermentation of intestinal bacteria, and metabolic processes involving S-adenosyl methionine. Blood methanol concentrations in healthy humans are estimated to range between 0.20 ± 0.035 and 5.37 ± 0.08 mg/L, and these values are reported to be 400–1000 times lower than the toxic concentration [16]. The World Health Organization (WHO) reports that the average adult receives 1.5–14 mg per day (mean 7.75 mg per day) formaldehyde via foodstuffs [17]. The half-life of formaldehyde in humans is 1–1.5 min, while stable blood and intracellular concentrations are estimated 2.6 mg/L (87 μM) and 12 mg/L (400 μM), respectively. Using real usage data and based on EFSA Scientific Panel on Food Additives and Nutrient Sources Added to Food analysis, APM use is estimated to contribute 0.5–9.7% of daily total endogenous and exogenous methanol exposure. At ADI dosages, an approximately 4 mg/kg formaldehyde burden arises with APM use, and this contributes only 0.3–0.4% of exposure to formaldehyde of endogenous and dietary origins [18]. For these reasons, APM is thought to be safe by world health authorities.

The safety of APM has been investigated by in vitro and in vivo studies. In the present study, we mainly explored in vitro cytotoxic, genotoxic damage potential and antioxidant/oxidant activity of APM in primary human whole blood cell cultures. Our finding showed that APM led to decreasing in cell viability in a concentration dependent manner. APM at concentrations greater than 25 mg/L significantly reduced cell viability. There have been previous studies of APM and its cytotoxic effects, but the results are inconsistent. Similar to our results, Horio et al. reported that cell vitality decreased in a concentration -dependent manner following exposure of PC12 cells to 0–8 μg/mL APM for 72 h, and that a severe decrease in cell vitality occurred at higher concentrations than 1 μg/mL APM [19]. In addition, Sonnewald et al. [20] detected that APM increased the release of LDH from cerebral granular neurons and 45Ca influx into the cell, and that it led to cell damage, in a concentration-dependent manner. The studies have shown that the cytotoxic effect induced by APM exposure may be associated with the oxidative stress [21]. Because formic acid, the final product of methanol metabolism, causes hypoxia at the cellular level due to its inhibition of cytochrome oxidase, the terminal member of the mitochondrial electron transport chain, and results in oxidative stress. Therefore, methanol is an aspartame metabolite that is a toxicant and causes systematic toxicity [22].

Due to the concerns that oxidative homeostasis can be adversely affected by methanol metabolism occurring with APM use, studies have also investigated oxidative homeostasis in various organs in association with APM use. In the current study TAC and TOS levels did not significantly change in the cells treated with APM. However, rat studies have reported an increase in oxidative stress characterized by increased lipid peroxidation levels, and a decrease in levels of such antioxidant molecules as reduced glutathione and protein thiol, in erythrocytes [23,24], heart [25], brain [26,27], liver and kidney [27] tissues with APM use.

In our study, we determined an increase in CA frequency in human lymphocyte culture exposed to an IC50 concentration. These are in agreement with studies in which the genotoxic effect of APM has been investigated using the CA test. It has been reported that APM induces CA in mouse bone marrow cells at doses of 35 and 350 mg/kg bw [28] and in human lymphocyte culture at concentrations of 500, 1000, and 2000 μg/mL [29]. At the same time, publications have also reported that it leads to DNA damage in mouse bone marrow cells at 35 mg/kg at comet-tail DNA analysis due to DNA filament breaks [30], and that in its current form it is genotoxic. Conversely, previous studies have also reported that it did not increase sister chromatid exchange (SCE) [28,29] and was not mutagenic at Ames test analysis involving Salmonella typhimurium strains [29,30]. Otherwise, APM was described as a moderately genotoxic agent [31]**. **However, it was reported that no evidence of gene mutation induction in a series of bacterial mutation tests, and that although there was evidence of in vitro chromosome damage induction, this occurred secondary to cytotoxicity and did not involve primary DNA damage [32].

In addition to studies reporting a genotoxic effect of APM with in vitro tests, there has also been research into its carcinogenic effect under in conditions. Soffriti et al. [13] reported increased renal pelvis transitional cell carcinoma and peripheral nerve schwannoma in Sprague–Dawley rats following the administration of the APM in diet. In another study investigating the effect on risk of fetal malignancy following exposure to APM in fetal life, Soffriti et al. [33] reported an increased incidence of lymphoma/leukemia in male and female rats and of breast cancer in female rats. In addition, Combos et al. [34] reported a significant increase Ha-ras and c-myc oncogens and p53 tumor suppressor gene expressions in lenforeticular, bone marrow, and kidney tissues in rats exposed to dosages of 40, 200, and 2500 mg/kg. Among those studies reporting that the APM molecule has no carcinogenic effect, some have stated that it does not increase the risk of hematopoietic or brain cancer in humans [35], that it causes no increase in the development, growth, or fatality of pancreatic acinar carcinoma in mice [36], and that there is no association with the risk of neoplasm in humans [37].

In conclusion, the result of this study demonstrates that APM has a moderate cytotoxic activity and causes no significant effect on antioxidative/oxidative activity on human blood cells. Moreover, APM is potential genotoxic agent in human blood cells. Thus, usage of APM should be monitored and limited due to its adverse effects. Our study will promote further research related to the safety of APM.

## References

[ref0] (2015). High intensity sweeteners chemicals structure, properties and applications. Natural Science and Discovery.

[ref1] (2010). Gain weight by “going diet?” Artificial sweeteners and the neurobiology of sugar cravings:. Neuroscience.

[ref2] (1983). FDA approves aspartame as soft-drink sweetener.

[ref3] (2006). Results of long-term carcinogenicity bioassay on Sprague-Dawley rats exposed to aspartame administered in feed. Annals of the New York Academy of Sciences.

[ref4] (2007). Aspartame: a safety evaluation based on current use levels,  regulations, and  toxicological  and  epidemiological studies.

[ref5] (2017). Chronic aspartame intake causes changes in the trans-sulphuration pathway, glutathione depletion and liver damage in mice. Redox Biology.

[ref6] (2002). Human receptors for sweet and umami taste. Proceedings of the National Academy of Sciences of the United States of America.

[ref7] (2016). u2d77 S. Artificial sweeteners as a sugar substitute: Are they really safe? u2d89ogy.

[ref8] (2013). Long-term effect of aspartame on the liver antioxidant status and histopathology in Wistar albino rats. Biomedicine & Preventive Nutrition.

[ref9] (2017). Aspartame and soft drink-mediated neurotoxicity in rats: implication of oxidative stress, apoptotic signaling pathways, electrolytes and hormonal levels.

[ref10] (2018). The possible protective effect of N-acetyl-L-cysteine and folic acid in combination against aspartame-induced cerebral cortex neurotoxicity in adult male rats: a light and transmission electron microscopic study. u2d73ogy.

[ref11] (2014). Synergistic effect of N-acetylcysteine and folic acid against aspartame- induced nephrotoxicity in rats. International Journal of Advanced Research.

[ref12] (u2d74). First experimental demonstration of the multipotential carcinogenic effects of aspartame administered in the feed to Sprague-Dawley rats. u2d6eives.

[ref13] (1997). Aspartame: metabolism and toxicity. Türkiye Klinikleri Journal of Medical Sciences.

[ref14] (u2d6d). Neurobiochemical alterations induced by the artificial sweetener aspartame (NutraSweet).

[ref15] (2015). Metabolic methanol: molecular pathways and physiological roles. Physiological Reviews.

[ref16] (1997). Guidelines for Canadian Drinking Water Quality: Supporting Documentation.

[ref17] (2014). Endogenous formaldehyde turnover in humans compared with exogenous contribution from food sources. European Food Safety Authority Journal.

[ref18] (u2d67). Aspartame-induced apoptosis in PC12 cells. u2d64ogy.

[ref19] (1995). Effects of aspartame on 45Ca influx and LDH leakage from nerve cells in culture. Neuroreport.

[ref20] (2006). The effect of aspartame metabolites on human erythrocyte membrane acetylcholinesterase activity. Pharmacological Research.

[ref21] (1991). and formic acid toxicity: biochemical mechanisms. Basic &Clinical Pharmacology& Toxicology.

[ref22] (u2d61). Prooxidative effects of aspartame on antioxidant defense status in erythrocytes of rats. u2d5bences.

[ref23] (2015). Effect of long-term intake of aspartame on serum biochemical parameters and erythrocyte oxidative stress biomarkers in rats. Comparative Clinical Pathology.

[ref24] (2016). Sheeladevi R. Aspartame induced cardiac oxidative stress in Wistar albino rats. Nutrition Clinique et Métabolisme.

[ref25] (2014). Biochemical responses and mitochondrial mediated activation of apoptosis on long-term effect of aspartame in rat brain. u2d58ogy.

[ref26] (u2d56). Effects of long-term administration of aspartame on biochemical indices, lipid profile and redox status of cellular system of male rats. u2d57ogy.

[ref27] (2010). Article ID: 605921. In vivo cytogenetic studies on aspartame. u2d55.

[ref28] (u2d5e). Genotoxicity of aspartame. u2d51ogy.

[ref29] (2008). Genotoxicity testing of low-calorie sweeteners: aspartame, acesulfame-K, and saccharin.

[ref30] (2014). A review of the genotoxic and carcinogenic effects of aspartame: does it safe or not?. Cytotechnology.

[ref31] (2015). Aspartame: A review of genotoxicity data. u2d47.

[ref32] (2007). Degli u2d42, u2d43. Life-span exposure to low doses of aspartame beginning during prenatal life increases cancer effects in rats.

[ref33] (u2d44). The effect of aspartame administration on oncogene and suppressor gene expressions.

[ref34] (2006). Consumption of aspartame-containing beverages and incidence of hematopoietic and brain malignancies.

[ref35] (2017). u2d34 KA, u2d32 U et al. u2d35ogy.

